# Unveiling the gaps: A comprehensive, equity-focused observational examination of Emergency Department discharge

**DOI:** 10.1371/journal.pone.0331226

**Published:** 2025-08-28

**Authors:** Lynn G. Jiang, Maree Sats, Sowmya Sanapala, Madeleine Tierney, Brady Rippon, Natalie C. Benda, Radhika Sundararajan, Peter A.D. Steel

**Affiliations:** 1 Department of Emergency Medicine, Weill Cornell Medical Center, NewYork-Presbyterian Hospital, New York, New York, United States of America; 2 Department of Population Health Sciences, Weill Cornell Medical Center, NewYork-Presbyterian Hospital, New York, New York, United States of America; 3 College of Arts and Sciences Government, Cornell University, Ithaca, New York, United States of America; 4 School of Nursing, Columbia University, NewYork-Presbyterian Hospital, New York, New York, United States of America; Dayeh University, TAIWAN

## Abstract

**Objectives:**

Prior work has shown that Emergency Department (ED) discharge instructions are often incomplete and poorly comprehended , leading to adverse events. Given the critical importance of effectively communicating ED discharge instructions, we performed a comprehensive evaluation of current adult ED discharge practices at two hospitals compared to a conceptual composite gold standard.

**Methods:**

This prospective observational study was conducted at two large academic urban EDs. ED patients were purposively selected for observation to ensure diverse representation including non-English language preferring (NELP) patients. Research Assistants (RAs) observed all provider-patient interactions throughout the patient’s ED encounter, documenting all discharge-related discussions. Verbal discharge and discharge documentation content were compared to a conceptual “gold-standard” checklist developed from consensus national guidelines.

**Results:**

Between September 2023 to March 2024, a total of 175 adult patient ED courses were observed. The majority of verbal communication regarding ED discharge included ED diagnosis (87%), ED diagnostic results (87%), medications (81%) and follow-up plan (78%). Only half (54%) included ED return precautions. Discharge documentation content mirrored these findings, except for ED diagnostic results (69%) and ED return precautions (81%). Assessment of comprehension occurred during only 53% of discharges, with 46% of patients having the opportunity to ask follow-up questions. For NELP patients, 12% of verbal discharges and 55% of discharge documentation were not in the patients’ preferred language.

**Conclusion:**

Much of the recommended ED discharge content was provided to the patient. Divergent content gaps identified across verbal discharge and discharge documentation support the pragmatic value of bimodal discharge processes. Given the demonstrated impact of discharge instruction comprehension on improving patient adherence, important foci for future process improvement includes assessing patient comprehension and exploring feasible solutions to facilitate the use of preferred language discharge documentation instructions.

## Introduction

Emergency Department (ED) discharge is a pivotal transition of care where the ED team communicates vital information to patients regarding their ED evaluation and post-ED care plan. According to the American College of Emergency Physicians (ACEP) [1–4], Society for Academic Emergency Medicine (SAEM) and Agency for Healthcare Research and Quality (AHRQ) [5–7], effective ED discharge educates patients on their evaluation and treatment plan [8–10], facilitates post-ED care, and ensures patient understanding of these instructions [8,9,11]. Despite its importance, delivery of high-quality discharge is hindered by ubiquitous ED care challenges such as overcrowding [12], time constraints, and interruptions [13,14]. Additionally, from a patient perspective, discharge can represent the end of an exhausting unanticipated healthcare encounter, a time of suboptimal information comprehension [1,5,6].

ED discharge represents a high-risk transition of care (TOC) and current ED discharge processes do not meet recommended national guidelines [2,3,15,16]. Prior work shows the majority of patients do not understand their ED discharge instructions [1,17–20] and poor comprehension correlates with poor care-plan adherence [21], contributing to high ED return rates and adverse clinical events [3,6,7,22,23], These deficiencies disproportionately affect vulnerable populations, including those with non-English language preference (NELP) [3,24–27]. Verbal ED discharge instructions alone are insufficient and lead to deficient patient comprehension on diagnosis, care plans, and return precautions [1–3]. While many institutions provide written ED discharge documentation, these documents are often incomplete and written in too technical language or at too high a literacy level [4,24,28–30]. A pilot study at Weill Cornell Medical Center (WCMC) ED in 2022 identified less than 50% of key written discharge documentation and verbal discharge content was conveyed to patients during ED discharge, similar to findings from prior published work.^8^ Overall, providers inadequately assess patient comprehension at the time of ED discharge, leading to poor patient recall and understanding [2,31]. These deficiencies disproportionately affect vulnerable populations, contributing to increased rates of poor medication and follow-up appointment adherence [3,24] as well as increased ED return visits and hospitalizations [25–27].

Similar to inpatient discharge [32]. the ED discharge process involves multiple interactions with multiple care team members throughout a patient’s encounter and typically culminates in a bimodal (written, verbal) process to “close” the encounter. The majority of prior work examining ED discharge has focused on individual aspects of this multistep process (e.g. discharge documentation quality, access to follow-up appointments), and to our knowledge there is no prior work on the ED discharge process that examines all discharge-related communication across all care-team members and modalities during a patient’s ED stay [8,28,33]. Furthermore, most studies evaluating the discharge process actively exclude or fail to meaningfully engage particularly vulnerable populations such as older adults and NELP patients [34–37], limiting current understanding of ED discharge process challenges for these patient populations.

We aimed to close these knowledge gaps by observing all ED discharge interactions and examining both verbal discharge and discharge documentation, recognizing that closing the ED encounter is a multistep bimodal process. We observed all verbal discharge-related interactions occurring with physician and nursing providers, when nursing verbally reviewed discharge documentation with the patient, as well as reviewing discharge documentation directly. We also evaluated a diverse group of patients, including those with NELP. Identifying and understanding the deficiencies currently present will allow us to address and improve the ED discharge process going forward, particularly for some of our most vulnerable patient populations.

## Methods

### Design and setting

We conducted a prospective cross-sectional observational study between September 2023 and March 2024 at an urban academic health system’s two demographically diverse EDs with approximately 113,000 adult visits annually. This study was deemed exempt from Human Subjects Research approval by the Weill Cornell Medicine Institutional Review Board.

In both EDs, the discharge process is a shared responsibility between providers and nursing staff. Patients interact with both clinicians and nurses prior to leaving: providers initiate the discharge by discussing the discharge plan and reviewing key information, while nurses reinforce this information by reviewing the written discharge instructions and addressing remaining questions. At the academic teaching hospital study site, a patient’s care team is layered, typically comprising resident trainees, advanced practice providers (APPs), and attending physicians and a nurse. In this setting, provider-side discharge communication is often performed by residents or APPs under the supervision of attending. By contrast, the smaller community-based hospital site operates with a leaner staffing model. Each patient is typically cared for by a single attending physician and assigned nurse, with the provider-nurse dyad jointly responsible for all aspects of the discharge process—including clinical communication and logistical coordination. While the expectation of discharge process quality is the same at both sites, these staffing model and team composition differences shape how the process is operationalized.

### Selection of participants

We enrolled a purposive, convenience sample of adult ED patients; patients being evaluated for a primary psychiatric diagnosis, patients less than 21 years old, and patients admitted to the hospital were excluded. In order to ensure that all discharge-related interactions were observed, we excluded patients whose ED evaluation would last outside the RA shifts or for >12 hours (longer than the Research Associate shifts, precluding them from observing the patient’s entire evaluation) and NELP patients who spoke to ED providers in their preferred language without using a translator (e.g.: both patient and provider spoke the same non-English language). Patients being discharged by the investigators of this study and patients who did not consent to be observed were also excluded. Patients whose care extended beyond RA hours, even if their visit began during the enrollment window, were excluded from analysis.

In order to limit Hawthorne bias, front-line staff were informed that they would be observed for a general observational study on patient interactions, blinding subjects to the specific focus on discharge interactions. All staff were given the opportunity to opt out of observation. Patients were informed that observations were to collect information to improve the patient experience. After this information was provided, front-line staff and patients would provide verbal consent to be observed. Consent was documented by the Research Associates (RAs) in a secure, HIPAA-compliant tracking log at the time of enrollment. All RAs were trained to confirm consent was clearly granted and to document this in real time. This verbal consent process was reviewed and approved by the IRB as part of the study’s exemption determination. The need for further consent was waived by the Weill Cornell IRB as there was no change to the standard of care for patients due to being observed.

Observations were performed by Research Associates (RAs) physically embedded in both EDs. RAs were present in-person, 12 hours a day from 8:30am to 8:30 pm, 7 days a week. These hours aligned with peak median emergency department census and boarding volumes at both study sites, capturing the majority of patient encounters and clinical activity during the day. RAs would review the electronic health record (EHR) ED track board in real-time to identify potential eligible patients. In order to ensure a heterogenous sample representative of our overall patient population across both ED sites, we purposively sampled patients based on age, gender, preferred language and level of medical acuity. Each demographic characteristic was divided into a binary categorization – for age, 65 years or older versus under 65 years old; male versus female; English versus non-English language; and evaluation in the acute (ESI 1–3) versus less acute (ESI 4–5) medical bays of the ED. RAs would preferentially select individual patients based on the above demographic characteristics to ensure equitable representation by the end of study enrollment period. This was evaluated on a weekly basis; running totals of currently enrolled patients and their demographics were distributed to the RA group each week to guide them as to which demographic characteristics should be prioritized during the next week’s enrollment. As a result, some eligible patients were not observed in the interest of prioritizing the diversity of patients in the study.

### Data collection

Once an eligible patient consented to study participation, RAs went to the bedside and observed the entirety of the patient’s ED evaluation from the first patient-provider interaction until discharge, documenting all discharge-related discussions from all provider types (physicians, advanced practice providers, nurses). No direct patient interaction or intervention occurred, and no protected health information was recorded. All patient-provider interactions related to the discharge process were timed including discussion of ED diagnostic results, follow-up plans, and medication changes. Any interactions with providers who were not part of the primary ED care team (e.g.: consultants) or interactions not related to ED discharge were not recorded.

Data collected included patient demographic information, the ED environment at the time of the patient’s discharge, as well as the total length of all discharge-related discussions and whether these discussions were interrupted. Once verbal discharge was complete and the patient formally discharged from the ED, RAs also examined the discharge documentation provided to patients at the time of discharge. All collected variables are shown in Table 1. The verbal discharge and discharge documentation content was compared to a conceptual “gold-standard” checklist developed from consensus national guidelines (Table 1 in S1 Table) [8,10,38]. Data was recorded as “yes”, “no” and “N/A”. The option N/A would apply in circumstances where the variable in question was not a part of that patient’s expected discharge – for example, a patient did not have new medications prescribed or have any medications changed, thus did not need a medication discussion in their discharge instructions. All data was recorded and stored in a centralized, HIPPA-compliant data entry system, Research Electronic Data Capture (REDCap) [39].

**Table 1:** Collected data variables from verbal discharge and discharge documentation observations.

Data Point Elements	Details
Date	Date of ED visit for patient
Patient Demographics	MRN, gender, age, and preferred language
Day of the week	Day of the week of ED visit for patient
Medical History	Presence/Absence of documented dementia diagnosis
Time of Discharge	The time when patient was discharged
Discharge Location	Where the patient was discharged from (eg: shared room, private room, hallway, etc.)
Number and Length of Interruptions	The number and duration of interruptions that occurred during patient-provider interaction
Length of Interaction	The duration of the patient-provider discharge interaction
ED Discharge Location	Where in the ED was the patient discharged from (eg: specific ED bay area and campus)
Length of Stay in ED	Total length of stay in ED
ED Patient Volume	Total number of patients currently in the ED at the time of the patient’s discharge
Verbal Discharge Diagnosis	Discussion of the final ED diagnosis or diagnostic uncertainty with the patient
Verbal Discharge Results	Discussion of results from any ED lab testing, imaging, etc. with the patient
Verbal Discharge Follow Up	Discussion of setting up a follow-up appointment with a provider outside the ED (e.g. PCP, specialists)
Verbal Discharge Return Precautions	Discussion of the circumstances for when a patient should return to the ED
Verbal Discharge Medications	Discussion of any medications that were prescribed or any medication changes during the ED visit
Verbal Discharge Comprehension	Provider assessment of whether the patient understood their discharge instructions
Verbal Discharge Questions	Discussion if the patient has any follow-up questions regarding their discharge
Verbal Discharge Other Parties Involvement	Discussion with the patient if they would like any other parties (e.g. family members, caregivers) to be involved in their discussion of the discharge plan
Verbal Discharge Inclusion of Dementia Diagnosis	If the patient has documented diagnosis of dementia, the provider would discuss with the patient if other parties need to be involved in patient care
Verbal Discharge Language	If the provider discussed the discharge plan in a language that the patient can understand
Verbal Discharge Transportation	Discussion with the patient assessing whether they have planned transportation back home
Discharge Documentation Diagnosis	If the discharge documentation included the patient’s ED diagnosis
Discharge Documentation Results	If the discharge documentation included imaging or test results from their ED visit
Discharge Documentation Follow Up	If the discharge documentation discussed a plan for a follow-up appointment with a provider outside the ED (e.g. PCP, specialists)
Discharge Documentation Return Precautions	If the discharge documentation outlined the circumstances in which the patient should return to the ED
Discharge Documentation Medications	If the discharge documentation discussed any medications that were prescribed or medication changes during the ED visit
Discharge Documentation Language	Were the discharge instructions written in a language the patient can understand

To ensure high quality data collection, RA training included one-on-one sessions with the study investigators to ensure that all RAs knew what information to record and had a standardized approach to recording their observations. Internal quality checks were conducted at least weekly to validate the team’s data collection where two RAs observed the same patient and independently recorded their observations; their results were then compared for congruence. Thirty-eight of the total 175 encounters (21.7%) were independently observed and scored by two trained Research Associates (RAs). We calculated Cohen’s kappa for categorical variables to evaluate agreement between raters beyond chance. Kappa values ranged from 0.64 to 1 (S3 Table) across key discharge elements, indicating moderate to substantial agreement.

### Statistical analysis

Patient demographic information was summarized by frequencies (%) for categorical variables and median (IQR) for continuous measures. Differences in both verbal discharge and discharge documentation variables were examined between gender, age (<65; 65+), preferred language, number of interruptions (none; one or more), and patient volumes at discharge (<100; 100+) by Pearson’s Chi-squared test. Agreement in records between these reports and corresponding quality review values were assessed via Chi-squared test or Fisher’s exact test where appropriate. All results are reported at a significance level of p < 0.05. All analyses were performed using R software version 4.4.1 [40].

## Results

### Characteristics of the observed interactions

Between September 2023 to March 2024, a total of 175 patient interactions were observed. Of those approached to participate, no providers or patients declined to be observed. The average patient age was 63 years [23–101; STD 18.7]; additional patient demographic characteristics are shown in Table 2. Average time of discharge discussion was 2.77 minutes [1.34–5.41]. Only 8% of these interactions had an interruption; of these, the majority were one interruption lasting less than a minute. Discharge conversations primarily occurred in a shared room (43%) followed by a hallway (24%) and private room (17%). Total ED volume at times of discharge was less than 100 patients for 55% of the observations and over 100 patients for 45% of observations.

**Table 2:** Demographic characteristics of observed patient discharges.

	Total Patients (N = 175)
**Gender**	
Male	84 (48%)
Female	91 (52%)
**Age**	
Younger than 65	98 (56%)
65 + years old	77 (44%)
**Preferred language**	
English	102 (58%)
Spanish	31 (18%)
Chinese (Mandarin/Cantonese)	19 (10.9%)
Other	23 (13%)

**Fig 1:**
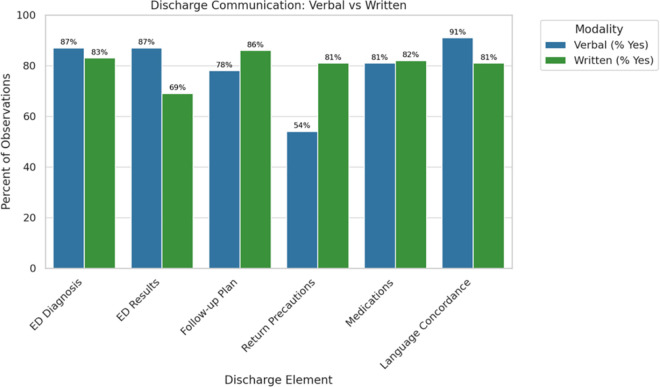
Examined verbal discharge and discharge documentation instruction content for all observed ED discharges.

**Fig 2:**
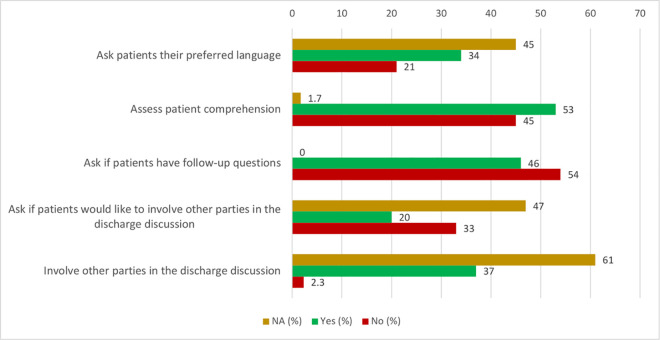
Patient-centered communication practices during verbal discharge instructions. Note: “N/A” was used when an element was not applicable to the encounter—for example, when no language discordance was present or accompanying individuals were already available.

The results from discharge content evaluation for both verbal discharge and discharge documentation for all observed discharges is shown in Figs 1 and 2. While most discharges included communication of the ED diagnosis, a small number of encounters were categorized as “Not Applicable” (N/A). These designations primarily reflected visits that were clinically straightforward—such as suture removals or medication refills—where the reason for the visit was self-evident, required no diagnostic workup, and did not prompt a formal diagnostic discussion. These encounters often occurred in our urgent care areas and typically involved no follow-up plan or testing, leading to “N/A” being applied to results and follow-up fields as well. Importantly, “N/A” was not used to denote diagnostic uncertainty. In our institutional practice, providers are expected to communicate a working diagnosis when diagnostic clarity is lacking (e.g., “chest pain,” “abdominal pain”), and such cases were captured as affirmative diagnosis disclosures in our coding. As such, the “N/A” responses reflect true clinical minimalism rather than ambiguity.

There was no difference in included discharge content for either discharge documentation or verbal discharge when analyzed by ED volume at the time of discharge (S2a and 2b in S1 Tables).

### Verbal discharge instructions

Almost 90% of discharges included discussion of ED diagnosis and ED diagnostic test results. Around 80% of discharges included discussion of the post-ED follow-up plan and any new prescriptions or changes to medication regimens. Only 54% of discharges discussed return precautions. 35% of patients were asked about their preferred language at the time of discharge. Our EHR system, however, often indicates what a patient’s preferred language is, especially if the patient has already been seen in our healthcare system, in which case a provider may already know the patient’s preferred language and thus not explicitly ask. This “N/A” group encompassed 45% of discharges. 94.1% of all discharges occurred in the patient’s preferred language. Most notably, 53% of discharges assessed patient comprehension of their discharge instructions and only 46% of patients were asked if they had follow-up questions.

ED diagnostic test results were discussed less frequently with women (82%) versus men (92%) (p = 0.040); ED diagnosis was discussed less often with older patients (65+) versus younger (<65 years old) patients (81% versus 92%, p = 0.026). Other parties were involved more often in discharge discussions for older patients (57% versus 9.2%, p < 0.001). Significant gaps in verbal discharge content were also observed with NELP patients. Return precautions were discussed in 63% of discharges with English speaking patients compared to 42% of NELP patients (p = 0.007) and patient understanding was assessed in 96% of English speakers compared to 85% of NELP patients (p = 0.003). While NELP patients were more likely to be asked about their preferred language (74%, p < 0.001), there was also increased frequency of discharge discussions not being in the patient’s preferred language during their discharge (12% versus 1% of English-speaking patients, p = 0.003). Anecdotally, RAs noted five interactions with inconsistent use of translator services (e.g.: a translator was used throughout most interactions but then not when the nurse went over discharge documentation with the patient just prior to leaving the ED).

### Discharge documentation instructions

Over 80% of discharge documentation included the ED diagnosis, a post-ED follow-up plan, new prescriptions or changes to medications, and return precautions. 69% of discharge documentation included the results of the ED evaluation.

There was no significant difference for any of the variables related to discharge documentation content by age or gender. When examining variables based on preferred language, however, there were several areas in which key content was included less frequently for NELP patients compared to English speaking patients. For NELP compared to English speaking patients respectively, discharge documentation instructions included ED diagnosis in 77% versus 88% (p = 0.011) of discharges, return precautions in 61% versus 91% of discharges (p < 0.001), and new prescriptions/medication changes in 74% versus 88% of discharges (p < 0.024). In addition, discharge instructions were written in the patient’s preferred language in 55% of discharges for NELP patients versus 99% of discharges for English speaking patients (p < 0.001).

## Discussion

This study offers a comprehensive examination of the gaps in current ED discharge processes by focusing on the ED discharge *process*, which involves multiple providers and various forms of communication across several different interactions over the course of a patient’s ED evaluation. Compared to prior work [3,8,41,42], this expansive approach, examining all discharge-related communication – both verbal provider-patient interactions and discharge document content – across all of the ED care team addresses ED discharge as a continuum, allowing a more thorough examination of the many components. Moreover, we included two typically vulnerable populations – older adults and patients with NELP. These groups face unique challenges in ED discharge care and communication yet are frequently underrepresented in similar research. By ensuring equitable representation, we were able to highlight critical areas for improvement to address some of their unique vulnerabilities.

While specific content areas (such as verbal discussion of return precautions, including ED evaluation results in discharge documentation) require improvement, our findings underscore the gaps in verbal versus written discharge information are not always concordant. This highlights the importance of both modes of information delivery in ensuring complete and effective communication of discharge information. Interestingly, despite the established links between ED overcrowding and care quality [12,43], we did not observe a difference in discharge content based on ED volume at time of discharge. Our analysis used total ED census (the total number of patients, including admitted patient “boarding”, present in the ED at the time) as a proxy for crowding. We believe this approach may best reflect the operational environment that shapes discharge communication than boarding burden alone, as all patients physically present contribute to staff workload, room turnover constraints and time pressure. However, future studies could benefit from a more granular approach that distinguishes between actively managed ED patients and boarders to clarify these dynamics.

The most consistent gap identified was ensuring that patients fully comprehend their discharge instructions and have opportunities to seek clarification, which is echoed by previous work [1,2,29,44,45]. Patient follow-up questions not only enhance patient understanding but promote active engagement in the discharge process, improving patient satisfaction and care-plan adherence [46–48]. Given additional communication challenges such as hearing loss and cognitive impairment in older adult populations [49–51], the importance of confirming comprehension is even more pronounced. While other parties were involved more often in discharge discussions for older patients, this was still only about half of the time, indicating area for improvement to include family members or caregivers in ED discharge processes.

Concern regarding patient comprehension of ED discharge instructions is particularly prominent with NELP patients. Prior work shows that NELP patients are at higher risk for poor medication and follow-up appointment adherence leading to increased ED return visits and hospitalizations [24–27]. We found increased rates of missing content in both verbal discharge and especially discharge documentation. Despite frequently identifying which patients preferred using a non-English language, more than 10% of discharges with NELP patients did not occur in their preferred language. Moreover, almost half of discharge documentation was not provided in the patient’s preferred language. While our institution provides 24/7 access to professional interpreter services across nearly all commonly spoken languages, RAs anecdotally observed that interpreter use was sometimes inconsistent during discharge, particularly when nursing staff reviewed written instructions. In several instances, there were perceived workflow challenges in engaging language services — such as needing to locate a translation iPad (which was frequently relocated within the ED) or call in a phone interpreter, both of which could introduce delays. This mirrors results seen in inpatient and pediatric discharges where 25–66% of NELP patients did not have a translator present during hospital encounters and 33%−50% reported lack of access to translated discharge documentation materials [52–56]. These findings emphasize the importance of optimizing interpreter access workflows and reinforcing expectations for their consistent use during all phases of ED discharge. With comprehension overall assessed less frequently in NELP patients, this suggests significantly higher risk and vulnerability in discharge care transitions for this already vulnerable population.

While we did not specifically examine the literacy level or medical jargon of discharge documentation, there may be a role for artificial intelligence to improve readability of discharge documentation materials, [57–59] which may also enhance patient comprehension [30]. Our results also highlight a specific need for communication interventions tailored to NELP populations, who may experience greater communication challenges affecting their overall understanding. We found deficiencies in consistent communication using patients’ preferred languages, despite having 24/7 translation services available – a gap that may be more prominent in the many health systems where such resources are not as readily available. To date, there is not an approved method to translate discharge documentation materials into non-English languages, explaining in part the gap in discharge documentation identified for our NELP patients. Some common conditions have pre-generated discharge materials embedded in the EHR in select languages (e.g., Spanish, Chinese); however, these templates are often generic, not tailored to individual patients, and frequently lack detailed or actionable instructions. They are also not easily editable by clinicians, which limits their utility in more complex or nuanced cases. In the absence of a centralized solution, anecdotally some providers resort to ad hoc approaches such as using Google Translate. Previous studies have explored the use of automated translation tools like Google Translate [60], but these tools often suffer from issues related to quality and accuracy, particularly when translating complex medical information. As a result, the majority of written discharge instructions are generated in English, even for patients who prefer a non-English language. As discharge documentation is intended for reference after the patient leaves the ED and their effectiveness is diminished if they are not in a language the patient understands, this is a crucial care quality gap that needs to be addressed to improve transitions of care in these populations.

More broadly, incorporating structured, patient-centered communication strategies into the ED discharge process—alongside deliberate opportunities for patients to ask questions and clarify uncertainties—can improve both patient comprehension and safety [29,46,61,62]. This is especially crucial in scenarios involving diagnostic uncertainty, which are common in emergency care. Studies show that a significant proportion of ED patients are discharged without a definitive diagnosis, contributing to confusion, anxiety, and unplanned return visits [33]. To support clearer communication in these high-uncertainty situations, tools like the Uncertainty Communication Checklist [63] developed by Rising et al., provide practical frameworks to help clinicians navigate ambiguous diagnoses while addressing patients’ needs and concerns [64]. Clear communication is essential when a definitive diagnosis is not established as this may present a unique and significant challenge to post-ED discharge care planning.

### Limitations

This study has several limitations. First, participants were a convenience sample of adult ED patients, which may not be fully representative of our broader ED population. We took a proactive, equity-focused approach to recruitment in order to achieve as representative and diverse a population as possible. Second, the study was conducted at two urban academic EDs, which may limit generalizability. Although the inclusion of two distinct sites – one a large academic tertiary center with layered teams, the other a smaller, community-based academic ED with leaner staffing—introduced meaningful variation in care team structure and discharge workflows, both sites operate within a single academic system. As such, they may benefit from greater staffing flexibility and support than many resource-constrained environments, where a single provider may be solely responsible for all aspects of care and discharge. These structural differences should be considered when interpreting the applicability of our findings to other settings.

Third, we excluded patients whose ED evaluations exceeded 12 hours, due to the requirement for RAs to observe the entire patient encounter. Although many of these excluded cases involved patients awaiting advanced imaging (e.g., MRI) with uncomplicated discharges following negative results, some may have involved complex care needs or atypical discharge communication dynamics. Furthermore, all data were collected between 8:30 AM and 8:30PM, when RAs were present. This time-bound sampling excludes many overnight encounters, which may differ due to patient characteristics, provider fatigue, or altered workflows. These limitations introduce potential selection bias and restrict our ability to evaluate discharge communication across a full 24-hour operational cycle. However, we note that the 8:30 AM to 8:30 PM timeframe corresponds with peak median ED census hours at both sites, capturing the majority of clinical volume and typical operational conditions. Despite these exclusions the demographic and clinical characteristics of the enrolled cohort were broadly representative of the overall ED population during the study period, supporting the relevance of our findings – with appropriate caution in generalizing to other operational models or timeframes.

Although we attempted to limit the Hawthorne effect by not informing providers or patients that discharge interactions were the specific focus of observation, we acknowledge that the awareness of being observed may have still influenced provider behavior. Providers may have altered their behavior simply due to the presence of an observer. This may have resulted in an overestimation of discharge communication quality.

We also recognize that while study results show potential areas of improvement to enhance patient comprehension of their ED discharge instructions, we did not directly measure patient comprehension or follow-up with patients to evaluate understanding or knowledge retention. Recognizing this limitation is particularly relevant for vulnerable NELP and older patients, who may face additional challenges to comprehension such as cognitive impairment or lower literacy levels. Only three patients in our study had a formal diagnosis of dementia, but the study was not powered to investigate cognitive impairment in detail.

Future research should address these limitations by including overnight and extended-stay patients and expanding to non-academic and resource constrained settings to better examine discharge communication in these high-risk contexts. Additional studies could also assess readability of discharge documentation instructions, which we did not examine in this study, as use of medical jargon or instructions written at too high of a literacy level also contributes to poor patient comprehension [30,65]. Furthermore, future work should explore the quality of the discharge process when the diagnosis remains uncertain—an increasingly common scenario in ED care that presents unique communication challenges and risks for poor patient outcomes.

## Conclusions

This novel prospective observational analysis of the multistep, bimodal ED discharge process can provide valuable insight into future opportunities for process improvement and improved post-ED outcomes. While gaps in the content of ED discharge instructions exist, the combination of verbal and written information allows for gaps apparent in one modality to be closed by the other. Most significant is the need for effective and standardized communication strategies to improve and confirm patient comprehension of ED discharge instructions. In order to enhance ED discharge quality for some of our most vulnerable populations, future research should further explore the unique needs of NELP patients, developing interventions to meet those needs in a combined verbal and written ED discharge process.

## Supporting information

S1 Plos Strobe. (DOCX)

S1 Table 1,2 Conceptual gold-standard ED discharge content as developed from consensus national guidelines. [8–10]. S1 Table, 2a: Inclusion of Verbal Discharge Communication Elements by Emergency Department Volume. 2b: Inclusion of Written Discharge Communication Elements by Emergency Department. S2 Table: Raw Dataset and Data Dictionary.
(DOCX)

S3 Table Cohen’s Kappa by Key Categorical Variable. (DOCX)
