# Supplementary material for: Unveiling the gaps: A comprehensive, equity-focused observational examination of Emergency Department discharge
Source: PLoS One. 2025 Aug 28;20(8):e0331226. doi: 10.1371/journal.pone.0331226 (PMC12393726; doi:10.1371/journal.pone.0331226)

**Supporting 1 - Table 1*:* Conceptual gold-standard ED discharge content as developed from consensus national guidelines.**[1, 2, 37]

| **Verbal Discharge Content** | **Details** |
| --- | --- |
| Verbal Discharge Diagnosis | Discussion of the final ED diagnosis or diagnostic uncertainty with the patient |
| Verbal Discharge Results | Discussion of results from any ED lab testing, imaging, etc. with the patient |
| Verbal Discharge Follow Up | Discussion of setting up a follow-up appointment with a provider outside the ED (e.g. PCP, specialists) |
| Verbal Discharge Return Precautions | Discussion of the circumstances for when a patient should return to the ED |
| Verbal Discharge Medications | Discussion of any medications that were prescribed or any medication changes during the ED visit |
| Verbal Discharge Comprehension | Provider assessment of whether the patient understood their discharge instructions |
| Verbal Discharge Questions | Discussion if the patient has any follow-up questions regarding their discharge |
| **Discharge Documentation Content** | **Details** |
| Discharge Documentation Diagnosis | If the discharge documentation included the patient’s ED diagnosis |
| Discharge Documentation Results | If the discharge documentation included imaging or test results from their ED visit |
| Discharge Documentation Follow Up | If the discharge documentation discussed a plan for a follow-up appointment with a provider outside the ED (e.g. PCP, specialists) |
| Discharge Documentation Return Precautions | If the discharge documentation outlined the circumstances in which the patient should return to the ED |
| Discharge Documentation Medications | If the discharge documentation discussed any medications that were prescribed or medication changes during the ED visit |


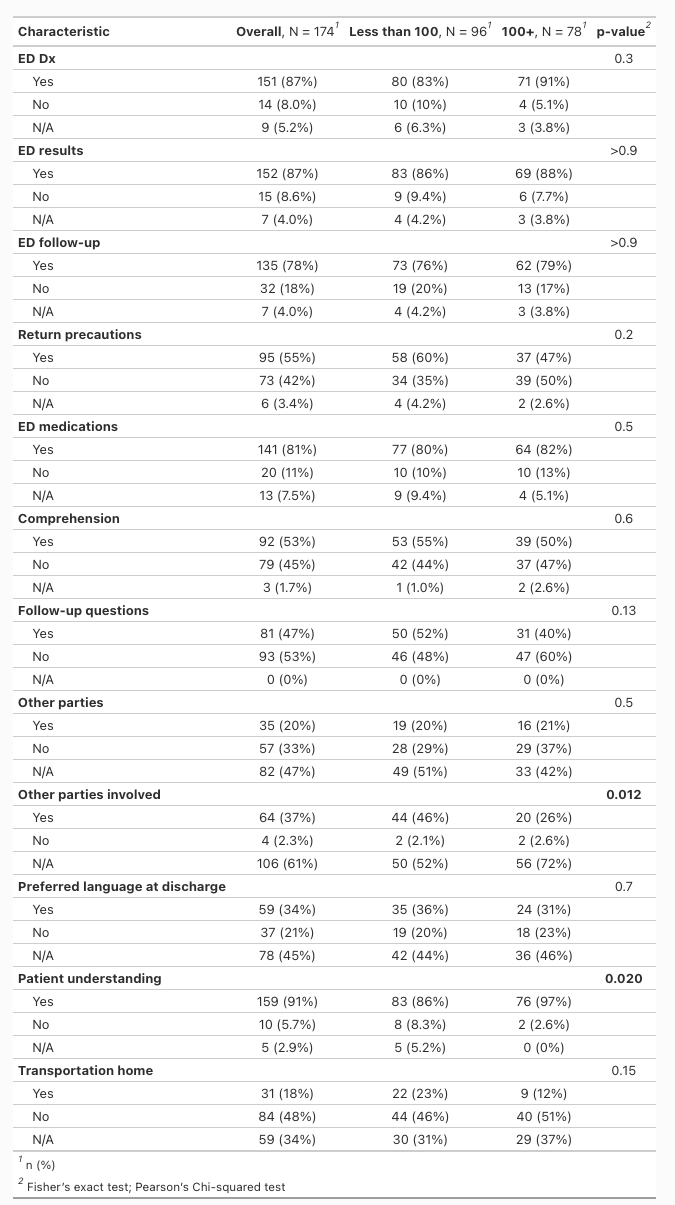
**Supporting 1 - Table 2a*:*** **Inclusion of Verbal Discharge Communication Elements by Emergency Department Volume**

**Supporting 1- Table 2b*:*** **Inclusion of Written Discharge Communication Elements by Emergency Department**


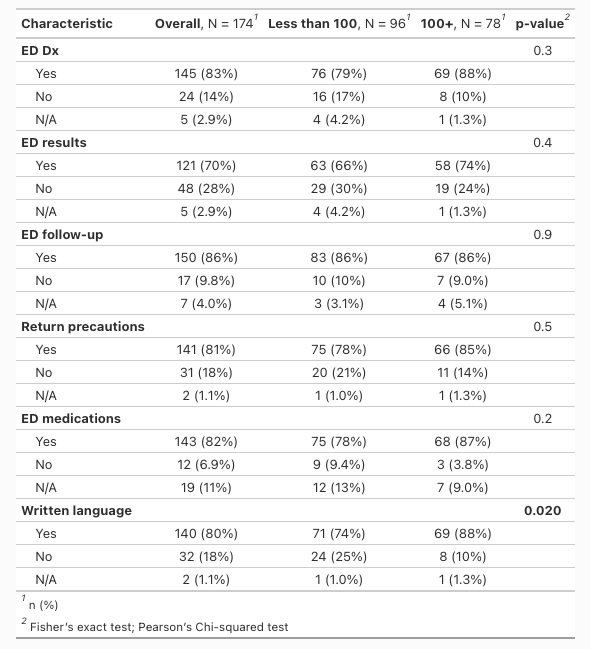

Supplement: S1 Table 1,2 — Conceptual gold-standard ED discharge content as developed from consensus national guidelines. [8–10]. S1 Table, 2a: Inclusion of Verbal Discharge Communication Elements by Emergency Department Volume. 2b: Inclusion of Written Discharge Communication Elements by Emergency Department. S2 Table: Raw Dataset and Data Dictionary. (DOCX) [file pone.0331226.s002.docx]
