# Supplementary material for: Unveiling the gaps: A comprehensive, equity-focused observational examination of Emergency Department discharge
Source: PLoS One. 2025 Aug 28;20(8):e0331226. doi: 10.1371/journal.pone.0331226 (PMC12393726; doi:10.1371/journal.pone.0331226)
Supplement: S3 Table — (DOCX) [file pone.0331226.s003.docx]

**Supporting 3 - Table 3: Cohen’s Kappa by Key Categorical Variable**

| **Categorical variable** | **Cohen’s Kappa** |
| --- | --- |
| *Verbal Discharge Variable* |  |
| ED Diagnosis | 1 |
| ED Results | 0.78 |
| ED Follow-up | 0.77 |
| Return Precautions | 0.9 |
| ED Medications | 1 |
| Check Comprehension | 0.64 |
| Follow-up Questions | 1 |
| Include Other parties | 0.64 |
| Use Preferred Language | 0.75 |
| Check patient understanding | 1 |
| Transportation Home | 0.64 |
| *Written Discharge Variable* |  |
| ED Diagnosis | 0.77 |
| ED Results | 0.95 |
| ED Follow-up | 0.87 |
| Return Precautions | 0.64 |
| ED Medications | 1 |
| Use Preferred Language | 1 |
